# Direct Sampling Mass Spectrometry Analysis for the Assessment of Wounds: A Systematic Review

**DOI:** 10.1111/iwj.70158

**Published:** 2025-03-24

**Authors:** Kanin Pruekprasert, Matthew Tan, Lauren Ford, Alun Huw Davies, Zoltan Takats, Sarah Onida

**Affiliations:** ^1^ Section of Vascular Surgery, Department of Surgery and Cancer Imperial College London London UK; ^2^ Department of Metabolism, Digestion and Reproduction Imperial College London London UK

**Keywords:** biomarker, mass spectrometry, metabolomics, ulcer, wound

## Abstract

Mass spectrometry is increasingly utilised in medicine to identify and quantify small biomarkers for diagnostic and prognostic purposes. Conventional mass spectrometry, however, requires time‐consuming sample preparation, hindering its clinical application. Direct sampling mass spectrometry, which allows for direct analysis of patient samples with minimal preparation, offers potential for clinical use. This systematic review examines the utility of direct sampling mass spectrometry for the assessment of external wounds and explores its translational applications in wound care. Out of 2 930 screened abstracts, six studies were included employing various direct sampling mass spectrometry technologies. These studies focused on burn wounds (*n* = 3), pressure ulcers (*n* = 2), and acute surgical wounds (*n* = 1). Both targeted and untargeted molecular profiling methods were used to examine biomarkers related to inflammatory and healing processes, including various proteins, lipid species, and other metabolites. Direct sampling mass spectrometry was found to complement conventional methods such as histology, providing additional insights into the spatial localisation and accumulation of metabolites within wounds. Additionally, imaging techniques equipped with this technology can spatially map wound surfaces and reveal dynamic changes in wounds as they age or progress through different healing processes, with specific metabolite and protein accumulations potentially aiding in prognostication.

**Summary**

- Direct sampling mass spectrometry is a novel technology offering rapid molecular profiling that potentially complements traditional diagnostic methods.
- This systematic review assesses the application of direct sampling MS in evaluating external wounds, encompassing six studies that focus on burn wounds, pressure ulcers, and acute surgical wounds.
- Direct sampling MS can identify molecular markers linked to various stages of wound healing, with imaging techniques providing spatial information across different wound areas.

## 1 Introduction

Mass spectrometry (MS) analytical platforms are increasingly used in the evaluation of a wide range of medical conditions, ranging from clinical laboratory [1] to intraoperative guidance in surgical procedures [2]. These platforms allow for the identification of alterations in levels of small molecules, including metabolites, proteins, and lipids, which may be related to disease processes, providing both diagnostic and prognostic information related to patient presentations. Conventional MS technology has been applied in studies analysing human wounds and skin samples in clinical laboratory settings [3, 4, 5, 6, 7, 8], however, its translational applications in real clinical settings are limited by the time‐consuming sample preparation required prior to MS analysis. These processes, including sample preparation and extraction, hinder the acquisition of quick or real‐time information, which is often necessary for clinicians to make contemporaneous management decisions.

Direct sampling MS comprises a wide range of MS techniques where the analysed samples are introduced directly to the mass spectrometry without chromatographic separation. This therefore allowing quicker analysis, especially for ambient MS technology where the ionisation process occurs outside of the mass spectrometer, enabling direct analysis of the samples of interest in their native state at ambient environment [9]. The minimal sample preparation enables ambient MS to provide rapid, in vivo and ex vivo molecular spectra of biological samples, and offers a promising potential for decision‐making in clinical settings, where quick, real‐time information is required.

**FIGURE 1:**
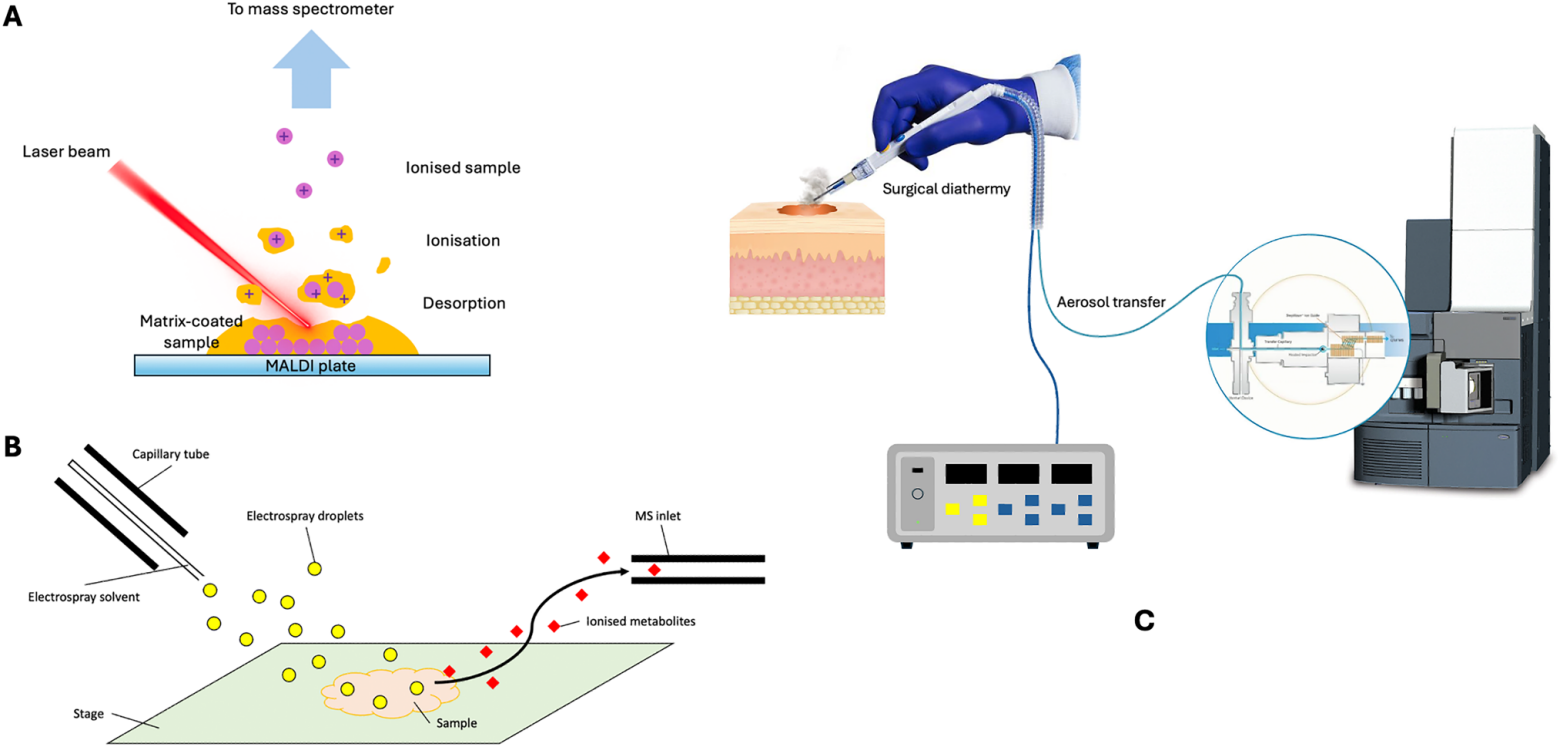
Operational mechanisms of the three direct sampling MS techniques. (A) Matrix‐Assisted Laser Desorption/Ionisation Mass Spectrometry (MALDI‐MS) applies a laser beam to ionise sample mounted with matrix, producing ions for analysis. (B) Desorption Electrospray Ionisation Mass Spectrometry (DESI‐MS) sprays charged solvent droplets onto a sample to ionise molecules directly from the surface. (C) Rapid Evaporative Ionisation Mass Spectrometry (REIMS) captures ionised molecules generated by rapid thermal evaporation during tissue ablation.

The earliest example of direct sampling MS technology dates back to the early 1960s when laser desorption techniques were applied to generate ions for analysis. This was later combined with the use of a solid or a liquid matrix to preserve larger molecules introduced in the late 1980s, resulting in the development of Matrix‐assisted Laser Desorption/ionisation Time of Flight MS (MALDI‐TOF‐MS) platforms [10, 11]. MALDI‐MS typically requires vacuum environment for effective ionisation, which differentiates it from ambient MS techniques. An ambient variant known as Ambient Pressure MALDI (AP‐MALDI) was later developed to enable sample ionisation at atmospheric pressure, however, the approach generally exhibits lower sensitivity and signal stability [12, 13]. It was only in the last two decades that a number of new ambient MS techniques were developed. This includes, for example, Direct analysis in real time (DART) and Desorption Electrospray Ionisation MS (DESI‐MS), which apply ionising gas or liquid solvent spray directly to samples to produce gaseous ions for MS analysis [14, 15], and Rapid Evaporative Ionisation MS (REIMS) which gathers molecular ions produced by rapid thermal evaporation of biological tissues [16], and many other techniques with variation of ionisation methods (Figure 1).

Wounds represent a major clinical, quality of life and financial burden to those affected and to society [17]. For the assessment and classification of wounds in clinical settings, most healthcare professionals rely on their own clinical experience or tools such as various scoring systems, which are highly operator dependent and also largely based on subjective observation to classify and quantify unhealthy characteristics of wounds from external appearance [18, 19, 20]. Direct sampling mass spectrometry technology allows for quick molecular spectral acquisition and can potentially provide objective information for these purposes by the recognition of specific spectral characteristics, as well as the identification of specific biomarkers. This can potentially lead to clinical decision guidance and development of wound care pathways. This concept has also been successfully demonstrated in human tissue with REIMS coupled with surgical diathermy, known as iKnife, for the rapid recognition of cancerous tissues in both in vivo and ex vivo samples as well as the detection of surgical margins [21, 22, 23].

This systematic review aims to investigate the use of direct sampling mass spectrometry for the evaluation of external wounds and to provide a qualitative summary of the findings and the value of the implementation of this technology for external wound evaluation in clinical practice.

## 2 Methods

The systematic review was conducted and reported according to the PRISMA guidelines [24]. The review protocol was prospectively registered on PROSPERO (ID: CRD42023398352).

### 2.1 Search Strategy

MeSH headings and other keywords pertaining to patients with external wounds and direct sampling mass spectrometry evaluation were used as search terms. The following search algorithm was developed to search the Medline and EMBASE bibliographic databases from 1946 to July 18, 2024. (Supporting Information: File 1).

A systematic three‐step process was used to identify relevant articles. Duplicates were removed by an automated system on an online systematic review platform (Covidence). After excluding all duplicates, two reviewers (K.P. and M.T.) independently performed an abstract screen to identify potentially relevant articles. The shortlists from both reviewers were then combined, and any disagreements were resolved in person by consensus. Lastly, both reviewers performed a full‐text review on all potentially relevant articles to ensure they met the inclusion and exclusion criteria. The references of the included articles were then screened for additional relevant articles. Unresolved disagreements were submitted to a third reviewer (S.O.) for resolution.

### 2.2 Inclusion and Exclusion Criteria

Any primary study (randomised control trial, prospective cohort study, retrospective cohort study, case series) reporting on the use of direct sampling MS of any techniques (e.g., matrix‐assisted laser desorption ionisation mass spectrometry (MALDI‐MS), direct analysis in real time (DART), desorption electrospray ionisation mass spectrometry (DESI‐MS), atmospheric pressure solid analysis probe, low temperature plasma (LTP), dielectric barrier discharge ionisation, extractive electrospray ionisation, paper spray ionisation (PSI), and rapid evaporative ionisation mass spectrometry (REIMS), etc.) for diagnostic and/or prognostic evaluation, and/or the therapeutic management of external wounds was included. Studies not applying direct sampling mass spectrometry techniques, not reporting application of these techniques on external wounds, not reporting original research (e.g., systematic review, narrative review, meta‐analysis), not reported in English, conference abstracts, case reports, and animal studies were excluded.

### 2.3 Data Extraction

Data extracted included study design, inclusion and exclusion criteria of participants, techniques of direct sampling mass spectrometry used, types and the number of wounds included, methods of wound tissue sampling, main findings from the use of direct sampling mass spectrometry including the comparison to other reference standards, and overall findings of the study.

### 2.4 Risk of Bias Assessment

The quality of the included studies was assessed by the QUADAS‐2 tool, which evaluates four primary domains including patient selection, index test, reference standard, and patient flow [25]. In this systematic review, the reference standard is defined as any standard assessment tools for clinical wound assessment or specific gold standard methods of compound identification and quantification corresponding to those employing direct sampling mass spectrometry technologies for analytical investigations. The full details of all domain assessments are provided in Supporting Information: File 2.

## 3 Results

### 3.1 Study Characteristics

**FIGURE 2:**
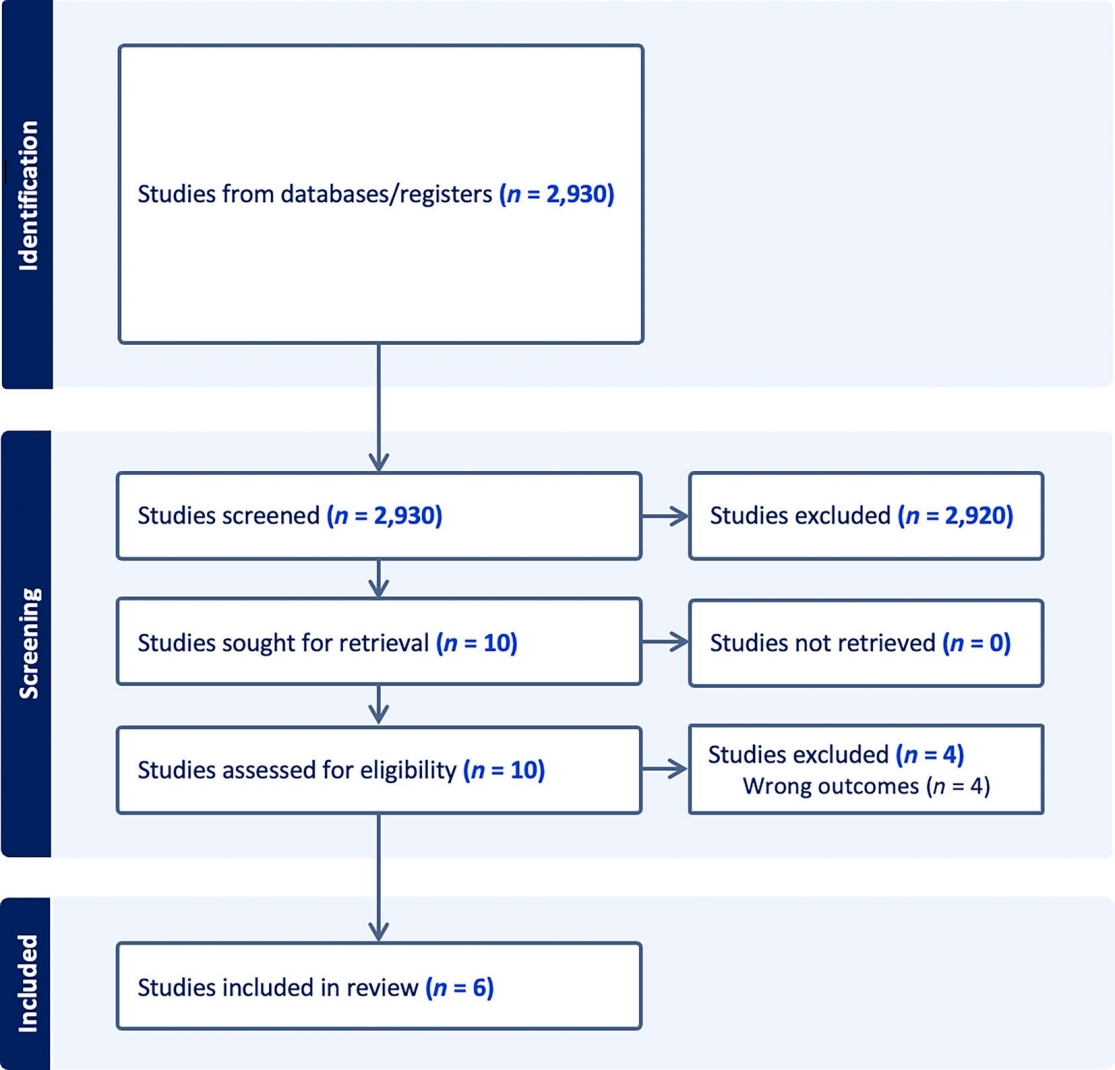
Prisma diagram.

A total of 2 930 articles were identified through the literature search. After applying predefined inclusion and exclusion criteria, 2 920 studies were excluded during the initial screening, primarily due irrelevant study focus or the lack of direct sampling MS application. Following this initial screening, 10 studies were selected for full‐text retrieval and assessed for eligibility. Upon further evaluation, four of these studies were excluded because their primary focus did not directly address external wound evaluation (e.g., studies only focused on microbiome identification without any direct relation to wound analysis). Consequently, six studies published between 2011 and 2022 were included in the final analysis (Figure 2).

These six studies utilised various direct sampling MS techniques, including MALDI‐TOF‐MS (*n* = 4), DESI‐MS (*n* = 1), and REIMS (*n* = 1), applying both targeted and untargeted molecular profiling methods. They examined a wide range of biomarkers, including proteins, lipid species, and metabolites, with potential relevance to wound inflammation or healing status. Of the six studies, three were performed on burn wounds [26, 27, 28], two on pressure ulceration [29, 30], and one remaining study was on acute surgical wounds [31]. A summary of findings from these studies is provided in Table 1.

**TABLE 1:** Summary of all included studies.

Study	Direct sampling MS technology used	Type of wounds	Method to obtain samples	Study design	Key findings from direct sampling MS
Taverna 2011 [29]	Autoflex II MALDI‐TOF (Bruker Daltonics, Billerica, MA) for protein analysis UltrafleXtreme MALDI TOF‐TOF and APEX‐Ultra FT‐ICR (Bruker Daltonics, Billerica, MA) for lipid analysis	Pressure ulcers (*n* = 6) Healthy controls (*n* = 3)	Pressure ulcer—surgical excision Healthy control—breast cosmetic surgery	MALDI‐MS imaging to spatially evaluate protein and phospholipid signatures from pressure ulcers at different healing status compared to healthy tissue samples	Demonstrates the feasibility for the identification of proteins and phospholipids, and spatial mapping of the molecular disturbances within the wound microenvironment – Alpha‐defensins (HNP‐1, −2, and − 3) were spatially expressed differently depending on healing status of the ulcers – TYB‐4 as a protein to be more expressed in adjacent dermis and maturing wound bed in healing ulcers – Identification of lipid species (e.g., glycerophosphocholines, glycerophosphoglycerols, glycerophosphoinositols, and glycerophosphates), of which some are distinctly expressed in specific ulcer areas at different healing status
Taverna 2015 [30]	AutoflexSpeed MALDI TOF spectrometer (Bruker Daltonics, Billerica, MA, USA)	Pressure ulcers of different healing status (*n* = 15)	Surgical excision	MALDI‐MS imaging to evaluate pressure ulcers that remain stalled, stagnant, and unhealed versus healing pressure ulcers	Multiple proteins such as human defensin‐6 and calcium‐modulated proteins (calcyclin, calgranulin A and B, and calgizzarin), to be spatially and uniquely expressed in different ulcer locations under different healing conditions – Calcycline, calgranulin A and B, and calgizzarin were specifically expressed in hypertrophic epidermis of the healing ulcers, while located only at wound bed or adjacent dermis in stagnant samples. – In intermediate ulcers, calcycline was displayed in papillary dermis close to wound bed, calgizzarin displayed at the epidermis and papillary dermis, while calgranulins displayed across both hypertrophic epidermis and wound bed. – HD‐6 specifically displayed in wound bed of chronic stagnant ulcers, with low levels detected in healing samples.
Taverna 2016 [26]	AutoflexSpeed MALDI TOF spectrometer (Bruker Daltonics, Billerica, MA, USA)	Burn wounds (*n* = 21)	Surgical excision	MALDI‐MS imaging for proteomic evaluation of partial thickness burn tissues, at different time‐points starting from acute burn injury until hypertrophic scar formation up to 15 months after burn injury	Demonstrates the feasibility of MALDI‐MS imaging to identify and spatially map different molecular characteristics of burn wounds throughout the entire healing process, for examples: – HNP‐1 located within wound bed at early stages but poorly expressed in hypertrophic scar and normal skin. – TYB‐4 were highly expressed in ulcer edge dermis during acute response and later become patchy distribution within subsequent scar tissue, while barely detected normal skin. – Calcium binding proteins (e.g., calcyclin, calgranulin A, and calgizzarin) were localised within dermal area of skin edge at early stage. Meanwhile, for scar tissues, calcyclin was localised within upper dermis and calgranulin A was found to be in papillary dermis, while calgizzarin was expressed in both. In normal tissues, calcyclin was almost absent, calgranulin A was ubiquitously expressed, and calgizzarin was restricted only in epidermis.
Castellanos 2020 [31]	SolariX 7.0 T MALDI FT‐ICR MS (Bruker Daltonics Inc., Billerica, MA, USA)	Acute surgical wounds (number not specified)	Discarded tissue samples from reduction surgery	MALDI‐MS imaging to confirm results from TOF‐SIMS in the evaluation of acute surgical wound at 0, 48, and 96 h after wound induction in ex vivo human skin tissue samples	Observations from MALDI FT‐ICR MS imaging and TOF‐SIMS were in good agreement – Endogenous lipid species localised in discrete epidermal skin layers – Diminished cholesterol sulphate signal along the stratum corneum toward the epithelial tongue in re‐epithelialising skin
Cuddihy 2021 [27]	DESI‐MS imaging (Waters, Manchester, UK)	Burn wounds (*n* = 38)	Surgical excision	MALDI‐MS imaging to evaluate lactate dehydrogenase (LDH) activity in burn wounds compared to LDH staining	Areas of relative abundance high LDH staining in good agreement with the presence of high pyruvate and low lactate demonstrated in DESI‐MS imaging – Increased inflammation in burn tissue compared to healthy control according to LDH activities – Higher LDH activity as the wounds age, regardless of different burn aetiologies – Inflammatory cells were more abundant in regions expressing high aerobic LDH activity.
Yau 2022 [28]	REIMS (Waters, Wilmslow, UK)	Burn wounds (*n* = 3) Healthy control (*n* = 8)	Discarded tissue from surgical operation	REIMS to obtain metabolic profiles from burn wounds compared to healthy control	Clear separation of molecular spectra between wound tissue and healthy control in OPLS‐DA classification model

Abbreviations: ALDI‐MS: matrix‐assisted laser desorption/ionisation mass spectrometry, DESI‐MS: desorption electrospray ionisation mass spectrometry, FT‐ICR: Fourier transform ion cyclotron resonance, HD: human defensin, HNP: human neutrophil peptide, LDH: lactate dehydrogenase, OPLS‐DA: orthogonal partial least squares discriminant analysis, REIMS: rapid evaporative ionisation mass spectrometry, TOF: time‐of‐flight, TOF‐SIMS: time‐of‐flight secondary ion mass spectrometry, TYB‐4: thymosin beta‐4.

### 3.2 Quality Assessment of Studies

**FIGURE 3:**
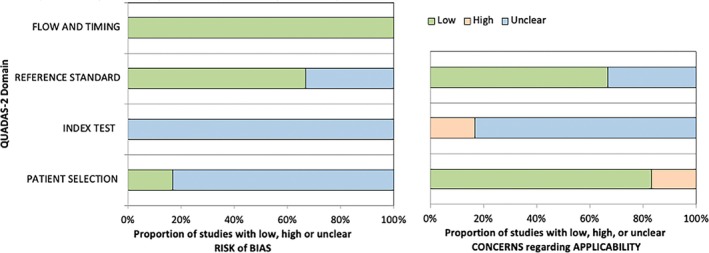
Outcomes of QUADAS‐2 study quality assessment.

The outcomes from the study quality assessment using the QUADAS‐2 are demonstrated in Figure 3, with further details elaborated in Supporting Information: File 2. For the risk of patient selection bias, most studies are classified as unclear risk due to the lack of details regarding patient recruitment. However, in terms of applicability, most studies included patients with external wounds relevant to the defined target population in this systematic review. The risk of bias and concerns of applicability in the index test domain were classified as unclear for most studies. These were due to the lack of clarity regarding blinding of assessors, as well as the concerns with the repeatability of this novel technology, which is currently being investigated. In the domain of the reference standard, the majority of the studies were categorised as having a low risk and concerns, except for two studies that relied on subjective assessment by clinicians.

### 3.3 Direct Sampling MS Technologies in Wound Assessment

#### 3.3.1 MALDI‐MS

MALDI‐MS was the direct sampling MS technique most studied in external wounds. MALDI‐MS imaging was used for spatial mapping of molecular disturbances within the microenvironment of external wounds. It has also been employed to identify various biomarkers potentially associated with wound age and healing status.

Taverna, et al. applied MALDI‐MS imaging in a study of six pressure ulcers, demonstrating unique spectral profiles within specific areas of the wounds. When compared to three healthy control tissues, the results demonstrated higher spectral intensities in the mass/charge (*m/z*) range of 2 500–4 000 displayed in wound bed, *m/z* signal of 4 965 observed in dermis, *m/z* range of 4 000–6 000 in adjacent dermis, and *m/z* range of 9 900–12 000 in hypertrophic dermis. Analysis of these different molecular spectra led to the identification of several proteins belonging to the alpha‐defensins family (human neutrophil peptide [HNP]−1, −2, and − 3) as potential markers expressed in the wound beds exhibiting little or no evidence of healing, and thymosin beta‐4 as a favourable marker in adjacent dermis and maturing areas of the wound beds [29].

**FIGURE 4:**
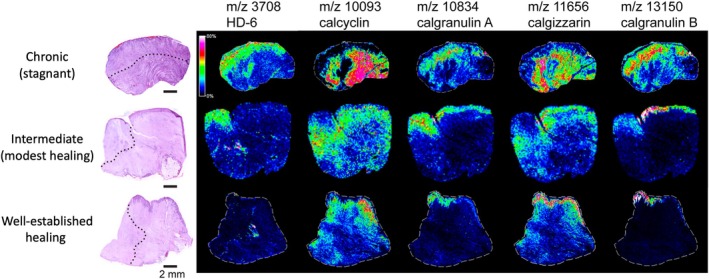
Pressure ulcer samples with different healing conditions, visualised using Matrix‐Assisted Laser Desorption/Ionisation Mass Spectrometry Imaging (MALDI‐MSI); (adapted from Taverna et al. [30] © American Chemical Society. This adaptation is unofficial). The left column displays histological staining of pressure ulcers. Dotted lines delineate focal regions of interest within each sample, with areas above or to the left indicating ulcer bed, and those below or to the right indicating adjacent dermis. The remaining columns present ion density maps of human defensin 6 (HD‐6) and four calcium‐modulating proteins (calcyclin, calgranulin A, calgizzarin, and calgranulin B) with relative ion intensities indicated by the colour bar, demonstrating their distinct localisation patterns under different healing conditions of pressure ulcers.

Using targeted methods, the same team further investigated the association of several calcium‐modulating proteins and their localisation according to the healing status of 15 stage IV pressure ulcers using MALDI‐MS technology. These proteins were expressed uniquely in different localisations under different healing conditions. Calcycline, calgranulin A&B, and calgizzarin were specifically displayed in hypertrophic epidermis of the healing ulcers, while they were located only in the wound bed or adjacent dermis in stagnant samples. In intermediate ulcers, calcycline was displayed in papillary dermis close to the wound bed and calgizzarin at the epidermis and papillary dermis, while the calgranulins were expressed across both the hypertrophic epidermis and throughout the wound bed. The authors also demonstrated human defensin 6 (HD‐6) to be specifically found in the wound bed of chronic stagnant ulcers, while low levels were found in healing samples (Figure 4) [30]. The change in proteomic profiles with wound age was demonstrated in burn wounds in another study from the same group examining 21 wound samples. HNP‐1 signals were highly observed in the wound bed when examined at day 3, 6 and 11, while poorly expressed in hypertrophic scar and normal skin. Thymosin beta‐4 was highly distributed in the dermis at wound edge during acute response and remains distributed in scar tissue, while barely detected in normal dermis. The three calcium modulating proteins, calcyclin, calgranulin A, and calgizzarin, were also expressed in specific patterns depending on the wound age, with possible link to inflammatory cell infiltration and fibroblast activity in tissue remodelling process, which were also confirmed by immunohistochemistry [26].

MALDI‐MS has also been employed for lipidomic studies in acute wounds by culturing healthy tissue biopsies to obtain ex vivo epithelised skin samples. MALDI Fourier‐transform ion cyclotron resonance mass spectrometry (MALDI FT‐ICR MS) was shown to be able to locate cholesterol sulphate, and specific fatty acids (FA 24:0 and 26:0) in stratum corneum with good agreement to the standard immunofluorescence imaging and TOF‐SIMS. The experiments also demonstrated that cholesterol sulphate signal in the stratum corneum diminished from the wound edge toward the epithelial tongue, suggesting its potential as a biomarker for human skin re‐epithelisation [31].

#### 3.3.2 Ambient MS

**FIGURE 5:**
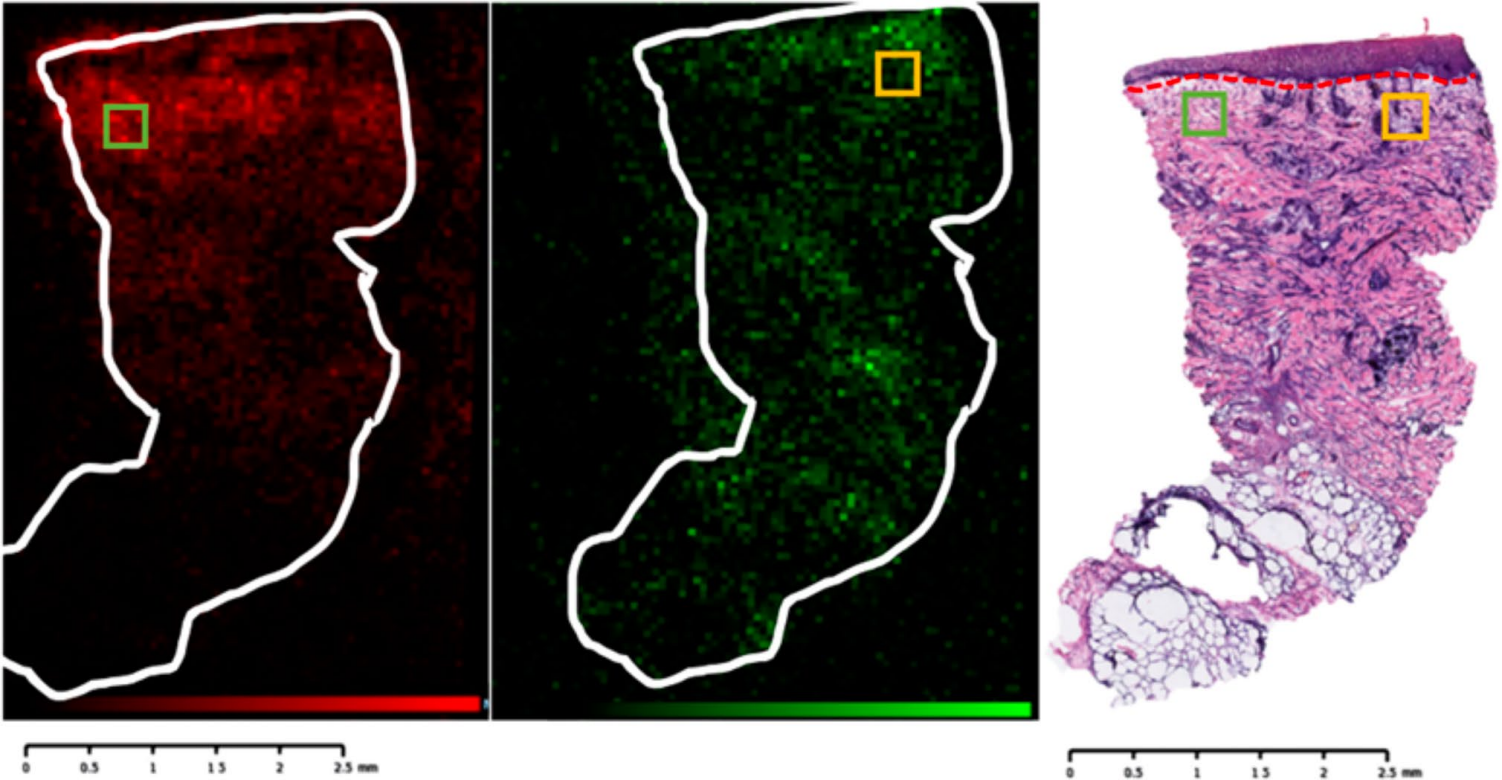
Burn injury sample visualised using Desorption Electrospray Ionisation Mass Spectrometry Imaging (DESI‐MSI), together with lactate dehydrogenase (LDH) staining (reproduce with permission from Cuddihy et al.) [27]. The left panel displays DESI‐MSI at *m/z* 89.0246, corresponding to lactic acid (shown in red), with higher expression in the upper left region (green box). The middle panel displays DESI‐MSI at *m/z* 87.0085, corresponding to pyruvate (shown in green), with increased expression in the upper right region (yellow box). The right panel displays an LDH‐stained section of the same tissue. Notably, regions with higher levels of LDH, an enzyme that catalyses the conversion of lactate to pyruvate, show spatial correlation with increased pyruvate expression (upper right). Conversely, regions with higher lactate expression (upper left) exhibit less LDH staining.

Ambient MS has also been employed in wound studies. DESI‐MS was used to assess lactate dehydrogenase (LDH) activity in burn tissue samples and healthy controls, compared to LDH functional staining method (Figure 5). DESI‐MS imaging demonstrated good correlation between areas of low lactate/high pyruvate distribution and the deposition of formazan in LDH staining tissue sections. The study showed that LDH activity was significantly increased in the middle and deeper regions of burns and also increased as the wounds aged, possibly due to CD4+ helper T lymphocytes and CD68+ macrophages infiltration into the dermis, which was observed on immunohistochemical staining [27].

In burn wounds, REIMS was used for ex vivo metabolic profiling compared to healthy skin controls. The Orthogonal Partial Least Squares Discriminant Analysis (OPLS‐DA) model created from molecular spectra obtained from REIMS demonstrated clear separation of wound tissue and healthy control spectra, with an area under the receiver operating characteristic curve (ROC) of 1.0. Additional targeted tandem liquid chromatography‐mass spectrometry (LC–MS/MS) lipidomics was performed in homogenised tissue samples revealing significant differences in specific lipid classes between healthy control and wound samples. In wound samples, free fatty acids, including monoacylglycerols, lysophosphatidylglycerol and lysophosphatidylethanolamines, were found to be at lower concentration while lactosylceramides and cholesterol esters showed higher concentrations when compared to healthy controls [28]. However, compound identification from REIMS analysis was not performed to validate and compare with LC–MS/MS results.

## 4 Discussion

This systematic review has revealed the feasibility of direct sampling MS technology to provide molecular spectra that allow for spatial localisation of external wounds, as well as the illustration of dynamic changes in molecular signature within the wounds that show diagnostic and prognostic potential. Various protein and lipid species identified from the wounds show specific differences when compared to healthy skin samples. This potentially allows for the differentiation between healing and non‐healing phenotypes, and further analysis of biological pathways corresponding to wound healing.

Conventional methods such as gel electrophoresis, western blot assay, enzyme‐linked immunosorbent assay, and isotope labelling have previously been used in metabolomic/proteomic wound research [32, 33]. However, these approaches are generally targeted, limiting their range of compound identification. This means they often miss low‐abundance metabolites, complex lipids, and certain post‐translational modifications that are important for a comprehensive profiling. Standard MS technology and nuclear magnetic resonance (NMR) spectroscopy offer metabolic profiling that can be employed for both targeted and untargeted compound identification to quantify metabolome/proteome expression and provide more information on post‐translational modification [34, 35, 36]. MS, in particular, can also be combined with conventional fractionation techniques, such as liquid chromatography (LC), to enhance compound separation and improve identification accuracy. Together, these platforms contribute to a more comprehensive understanding of complex biological changes and interactions within metabolic pathways and facilitate the discovery of new biomarkers. However, these metabolic profiling platforms require a lengthy process of sample preparation, therefore preventing rapid acquisition of molecular spectra, which greatly limits their translational potential in clinical practice.

Given that direct sampling MS technology requires less sample pre‐processing, this offers rapid molecular profiling compared to conventional MS methods. This can significantly enhance the translational potential of MS analysis in clinical practice, particularly in scenarios when quick or real‐time information is required. To integrate this technology into clinical care pathways, it is also mandatory for physicians to understand the limitations of each direct sampling MS method. For MALDI‐MS, sample preparation in terms of matrix mounting is still required prior to analysis [10], therefore not offering real‐time results. Electrospray ionisation techniques usually require methanol in a solvent in order to preserve large and non‐volatile molecules, such as lipids, from fragmentation [37, 38]. Therefore, such methods are more suitable to be applied to the collected samples rather than directly to patients. REIMS allows for direct in vivo application with minimal to no sample preparation required, therefore enabling the development of real‐time intraoperative evaluation platforms, especially when successfully combined to the already used surgical devices [16, 39, 40, 41]. However, both DESI‐MS and REIMS are generally limited to surface‐level analysis and are not yet fully integrated with comprehensive tandem MS capabilities. While targeted DESI analysis is possible with triple quadrupole mass spectrometers using a multiple reaction monitoring (MRM) table [42], this approach restricts the range of detectable features to those predefined in the MRM table. As a result, it can be less effective for hypothesis‐generating research and broad compound identification compared to conventional MS platforms, which often incorporate tandem MS to enable more detailed structural analysis and differentiation of isobaric molecules through sequential ion fragmentation [43].

The translational applications of direct sampling MS technology, particularly ambient MS, have been increasingly integrated into various areas of medicine in clinical settings. In clinical pharmacology and toxicology, several variations of electrospray ionisation mass spectrometry techniques have demonstrated the ability to detect and monitor the level of specific chemical agents in human bodily fluids [44, 45, 46, 47, 48, 49, 50]. In microbiology, MALDI‐TOF MS has been adopted for bacterial identification from diverse samples, often outperforming conventional methods in speed and accuracy [51, 52, 53], with REIMS also shows potential for rapid microbiome identification directly from samples [54, 55, 56, 57, 58]. In cancer research, MALDI‐MS, touch spray MS (TS‐MS), DESI‐MS, and REIMS have all been employed for tissue analysis and drug response evaluation, with REIMS, particularly the iKnife, showing the most promise [59, 60]. Multiple ex vivo and in vivo studies have demonstrated excellent accuracy of the iKnife for cancer identification [22, 23, 61, 62, 63]. REIMS has also been integrated with endoscopic devices for accurate real‐time identification of stomach and colonic malignant lesions during endoscopy [21, 40]. Furthermore, both DESI‐MS and REIMS have also demonstrated the ability to spatially identify tumour borders and resection margins from surgical samples [64, 65].

Similar to the aforementioned examples, direct sampling MS technology can potentially provide diagnostic and prognostic potential in the management of wounds. The identification of specific biomarkers from molecular signatures can lead to a better understanding of biological process and distinguish molecular characteristics specific to certain wound types and conditions, which may lead to development of more effective treatment. There have been several studies exploring wound metabolomics, including the associations to microbiomes identified in wounds using conventional metabolic profiling methods. Employing GC–MS, Thomas et al. demonstrated the feasibility to differentiate between arterial ulcer, ulcer border, and healthy skin areas by detecting volatile organic compounds collected from the application of skin patch, with further analysis demonstrating metabolic profiles associated with each sample area, bacterial colonisation, and patients' medications [66]. The same research group further demonstrated temporal and dynamic differences of metabolomes and microbiomes corresponding to the healing process of acute wounds for up to 4 weeks [67]. NMR has also been used in wound metabolic profiling. From venous leg ulcer exudates, Junka et al. identified metabolites associated with hypoxia and neutrophil metabolism, with metabolite identification enabling microbiome differentiation between *S. aureus* to other bacterial species, as well as the recognition of the patient's diabetic status [68]. Pressure ulcer tissues also displayed changes in metabolite accumulation according to the depth of tissue biopsy sections. Further metabolic pathway analysis found the link to selective amino acid metabolism with changes in some metabolites associated with specific bacterial genera identified by genome sequencing [69].

The characterisation of healing and non‐healing wound phenotypes can allow for prompt treatment decision and individualisation of treatment, enabling more efficient allocation of resource to individuals who will benefit the most. Bergner and Onida et al. evaluated the metabolic signatures in urine, serum, and ulcer fluid in 28 patients with venous leg ulceration (VLU) using both NMR and ultra‐performance liquid chromatography‐mass spectrometry (UPLC‐MS). The results demonstrated the ratio of carnitine to ceramide in serum to be predictive of ulcer healing with 100% sensitivity, 79% specificity, and 91% accuracy [70]. This finding, once validated, could potentially allow for prognostic guidance and future development of individualised treatment pathways according to VLU metabolic phenotype. The determination of wound age applying metabolomic methods has also been studied in animal model. Cao et al. demonstrated 92.6% accuracy of wound age estimation from four up to 48 h in rats using UPLC‐MS combined with machine learning algorithms to cluster the levels of 43 different metabolites identified from contused skeletal muscle tissues [71]. The potential of this technology to determine wound chronicity is to be explored further in humans, which may lead to specific or individualised treatment for example, characterisation of healing and non‐healing wounds, rapid identification and evaluation of microbiome and bacterial colonisation, detection of specific wound metabolic profiles to target topical therapy and indication of when sufficient debridement has been achieved to minimise removal of healthy tissue. Future research should focus on translational applications of direct sampling mass spectrometry technology into the wound care pathway, enabling its potential into clinical practice.

## 5 Conclusions

Direct sampling MS technology has proven its ability to be able to spatially localise molecular signatures of external wounds, and provide useful information to potentially improve the accuracy of conventional histological methods.

This novel technology has also shown promise in demonstrating dynamic changes in the metabolic and proteomic profiles of external wounds that can lead to diagnostic and prognostic potential. Diagnostically, direct sampling MS techniques have demonstrated the ability to accurately geographically map specific wound areas compared to conventional histological methods, as well as providing additional information on certain metabolites/proteins/lipids accumulated in different locations within the wounds. The technology can also be used to identify the unique differences in metabolic signature of certain wounds of different healing status. This may further permit differentiation between wounds that are healing and those that are not, allowing for individualisation of treatment according to specific wound phenotypes. To realise this potential in clinical practice, further research should focus on the translational applications of this technology and development of new, rapid, and minimally invasive platforms for wound evaluation into the clinical care pathway.

## Conflicts of Interest

The authors declare no conflicts of interest.

## Supporting information


Data S1.

## Data Availability

Data sharing not applicable‐no new data generated, or the article describes entirely theoretical research.
