# Supplementary material for: Direct Sampling Mass Spectrometry Analysis for the Assessment of Wounds: A Systematic Review
Source: Int Wound J. 2025 Mar 24;22(4):e70158. doi: 10.1111/iwj.70158 (PMC11932957; doi:10.1111/iwj.70158)
Supplement: Supplementary file 1 — Data S1. [file IWJ-22-e70158-s001.docx]

**Supplementary file 1: Search algorithm**

Search: ((ulcer*) OR (wound)) AND (((((((((((((((((((((((("rapid evaporative ionization mass spectrometry") OR (REIMS)) OR (iknife)) OR ("desorption electrospray ionization mass spectrometry")) OR (DESI-MS)) OR ("matrix-assisted laser desorption ionization mass spectrometry")) OR (MALDI-MS)) OR ("matrix-assisted laser desorption ionization time of flight mass spectrometry")) OR (MALDI-TOF-MS)) OR ("imaging mass spectrometry")) OR ("laser-assisted rapid evaporative ionization mass spectrometry")) OR (LA-REIMS)) OR ("direct sampling mass spectrometry")) OR ("direct analysis in real time")) OR (DART)) OR ("atmospheric pressure solid analysis probe")) OR ("laser ablation electrospray ionization")) OR (LAESI)) OR ("low temperature plasma")) OR (LTP)) OR ("dielectric barrier discharge ionization")) OR ("extractive electrospray ionization")) OR ("paper spray ionization")) OR (PSI))

**Supplementary file 2: QUADAS-2 evaluation**

| **Study** | **RISK OF BIAS** | | | | **APPLICABILITY CONCERNS** | | |
| --- | --- | --- | --- | --- | --- | --- | --- |
|  | **PATIENT SELECTION** | **INDEX TEST** | **REFERENCE STANDARD** | **FLOW AND TIMING** | **PATIENT SELECTION** | **INDEX TEST** | **REFERENCE STANDARD** |
| **Taverna 2011** | ? | ? | ? | ☺ | ☺ | ? | ? |
| **Taverna 2015** | ? | ? | ? | ☺ | ☺ | ? | ? |
| **Taverna 2016** | ? | ? | ☺ | ☺ | ☺ | ? | ☺ |
| **Castellanos 2020** | ? | ? | ☺ | ☺ | ☹ | ? | ☺ |
| **Cuddihy 2021** | ☺ | ? | ☺ | ☺ | ☺ | ? | ☺ |
| **Yau 2022** | ? | ? | ☺ | ☺ | ☺ | ☹ | ☺ |

**Signalling Questions for different domains** ☺Low Risk ☹High Risk ? Unclear Risk

**Patient Selection
Risk of Bias**
Was a consecutive or random sample of patients enrolled?
Was a case-control design avoided?
Did the study avoid inappropriate exclusions?

**Concerns regarding applicability**Do patients included in the study differ from those targeted in the review question in terms of type of target condition, demographic features, presence of differential diagnosis or co-morbid conditions?

**Index tests
Risk of Bias**Were the index test results interpreted without knowledge of the results of the reference standard?

**Concerns regarding applicability**Is there a concern that the Index test, its conduct, or interpretation (is the data scrutinised with sound statistical methods), differ from the review question?

**Reference standard
Risk of Bias**Is the reference standard likely to correctly classify the target condition?
Were the reference standard results interpreted without knowledge of the results of the index tests?

**Concerns regarding applicability**Is there a concern that the target condition as defined by the reference standard does not match the review question (histological evaluation of cancer and control samples)?

**Flow and Timing
Risk of Bias**Was there an appropriate interval between index test(s) and reference standard?
Did all patients receive a reference standard?
Did patients receive the same standard?
Were all patients included in analysis?
